# Fool's gold standard? Immunoperoxidase staining with the mouse monoclonal antibody (Clone 22C11) for detecting axonal pathology after traumatic brain injury

**DOI:** 10.3389/fnins.2025.1613172

**Published:** 2025-06-02

**Authors:** Guoxiang Xiong, Shanti R. Tummala, Akiva S. Cohen

**Affiliations:** ^1^Department of Anesthesiology and Critical Care Medicine, The Children's Hospital of Philadelphia, Philadelphia, PA, United States; ^2^Department of Anesthesiology and Critical Care Medicine, Perelman School of Medicine, University of Pennsylvania, Philadelphia, PA, United States

**Keywords:** brain trauma, amyloid precursor protein, immunoperoxidase staining, avidin, biotin

## Introduction

Traumatic brain injury (TBI) is a leading cause of morbidity and mortality in the United States (Coronado et al., 2011). Pathology and functional deficits resulting from TBI vary with the mechanical insults, but a common characteristic in the experimental as well as clinical realm is diffuse axonal injury (DAI). DAI is typically inferred from hemorrhages and axonal damage in white matter tracts detected with hematoxylin and eosin (H&E) staining and Palmgren silver impregnation (Adams et al., 1982, 1989). Furthermore, DAI can be confirmed with electron microscopy (Mierzwa et al., 2015; Ziogas and Koliatsos, 2018), tractography (Hayes et al., 2016; Nolan et al., 2021), NeuroSilver staining (Koliatsos et al., 2011; Xiong et al., 2023, 2024) and transgenic labeling (Hånell et al., 2015; Xiong et al., 2023).

It is hypothesized that disrupted transport in injured axons results in an accumulation of amyloid precursor protein (APP) at the sites of rupture. Using immunoperoxidase staining with a mouse monoclonal antibody (Clone 22C11) against the N-Terminus of APP, Gentleman et al. (1993, 1995) first demonstrated varicosities in white matter from TBI patients and interpreted them as axonal swellings. These 22C11-positive varicosities can be detected as early as 3 h after TBI (Gentleman et al., 1993, 1995; Sherriff et al., 1994a,b; Graham et al., 2004; Reichard et al., 2005; Hortobágyi et al., 2007; Johnson et al., 2013, 2016; Koch et al., 2020) and remain identifiable for months or even years after the initial insult (Chen et al., 2009; Johnson et al., 2013). Based on its wide application for more than three decades, immunoperoxidase staining with 22C11 is regarded as the “Gold Standard” for detecting axonal pathology after TBI (Johnson et al., 2013, 2016). However, the accumulation of APP in axons has never been unequivocally confirmed. Here, we summarize existing evidence that questions the specificity of 22C11 for APP and the validity of immunoperoxidase staining to reveal axonal pathology after TBI. We then provide an alternate interpretation of the observed varicosities and recommend a strategy for the accurate determination of TBI-induced neuropathology.

## Discussion

APP is a protein that is widely expressed in the brain (Del Turco et al., 2016; Xiong et al., 2023) and plays an important role in a variety of physiological functions (Hick et al., 2015; Müller et al., 2017). Antibodies specific to APP should therefore produce immunohistochemical staining patterns consistent with the expression of the protein in healthy i.e., non-injured brains. However, previous studies demonstrated negative staining with 22C11 in brains from control patients (See Johnson et al., 2013 for a review). Moreover, 22C11-stained varicosities were typically identified with an immunoperoxidase protocol, which makes it impossible to determine if these varicosities co-localize with any specific axonal marker (Gentleman et al., 1993, 1995; Sherriff et al., 1994a,b; Graham et al., 2004; Reichard et al., 2005; Hortobágyi et al., 2007; Johnson et al., 2013; Koch et al., 2020). A single group (Johnson et al., 2016) did attempt double immunofluorescent staining using 22C11 and the axonal marker spectrin N-terminal fragment (SNTF). However, using a porcine model of mild TBI as well as tissue from severe brain injured humans resulted in inconsistent results; thereby, failing to provide conclusive evidence for the claimed axonal identity of 22C11-positive varicosities. In addition, while varicosities are reliably reproduced in immunoperoxidase staining, they are not visible after immunofluorescent staining with 22C11 (Xiong et al., 2023). Lastly and most importantly, 22C11 produces a similar staining pattern in wild-type and APP knockout mice (Guo et al., 2012; Del Turco et al., 2016; Xiong et al., 2023), and stains out astrocytes (Chauvet et al., 1997; Young et al., 1999; Yasuoka et al., 2004; Xiong et al., 2023). Together, these observations indicate that 1) 22C11 does not specifically recognize APP, 2) an unknown protein this antibody actually binds is present on astrocytes but not necessarily in axons and 3) the varicosities observed with immunoperoxidase method after TBI are a product of the interaction of the reagents with the altered or newly expressed chemical components after TBI. Therefore, 22C11 is not the best or ideal marker for axonal damage after TBI.

**Figure 1:**
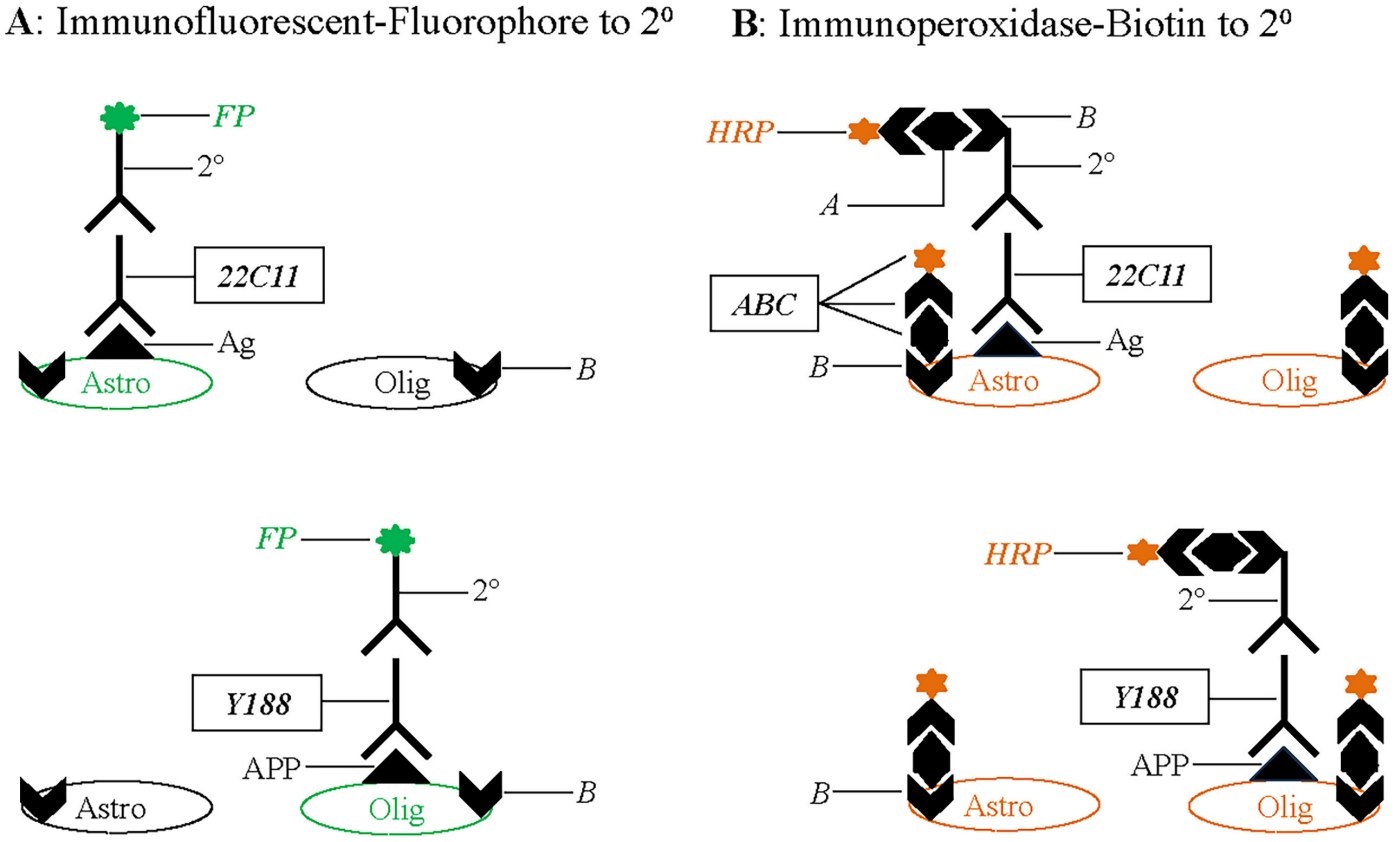
Summary diagram illustrating targets in white matter tracts revealed by immunofluorescent vs. immunohistochemical staining with the N-Terminal antibody (22C11) and C-Terminal antibody (Y188) for amyloid precursor protein (APP). **(A)** Immunofluorescent staining is performed using fluorophore (*FP*)-conjugated secondary antibodies (2°) that is visible under a microscope. An unknow antigen (Ag) in astrocytes can be specifically recognized by ***22C11***, and APP in oligodendrocytes by ***Y188***. **(B)** Immunoperoxidase staining is performed using biotinylated secondary antibodies that needs further incubation with the ***ABC*** kit before an enzymatic reaction for the horseradish peroxidase (*HRP*) contained in the staining kit. Avidin *(A)* in the kit will bind to biotin *(B)* that is conjugated to the secondary antibodies, resulting in specific immunostaining. Avidin can also bind to endogenous biotin *(B)* that is present in astrocytes and oligodendrocytes. Therefore, both glial groups can be coincidently stained by the ***ABC*** kit. Astro, astrocyte; Olig, oligodendrocyte.

What then are the varicosities reliably observed with immunoperoxidase staining? Careful comparison of immunoperoxidase to immunofluorescent staining suggests that avidin binding to endogenous biotin may be the source of the varicose signals. As illustrated in Figure 1, immunofluorescent staining (Figure 1A) of the primary antibody (22C11 or Y188, a validated specific antibody against the C-Terminus of APP) is accomplished with a fluorophore (FP)-conjugated secondary antibody (2°) that is species-specific for the primary antibody. Conversely, immunoperoxidase staining (Figure 1B) is performed using a biotinylated (or biotin-conjugated; *B*) secondary antibody that is also species-specific for the primary antibody, resulting in specific staining. Unlike FP-conjugated secondary antibodies that are readily visible under a light microscope, biotinylated secondaries can only be visualized after avidin binding via ABC incubation and an enzymatic reaction for horseradish peroxide (HRP) that is contained in the ABC staining kit. Avidin (*A*) in the ABC kit can also bind to endogenous biotin (Vitamin B7, a coenzyme for 5 carboxylases; Zempleni et al., 2009) in the tissue and generates a spurious (or non-specific) signal.

The interference from endogenous biotin to immunoperoxidase staining has been demonstrated for more than two decades (Bhattacharjee et al., 1997; McKay et al., 2004). However, it has been overlooked in the practice of ABC-mediated immunoperoxidase staining with 22C11 (Gentleman et al., 1993, 1995; Sherriff et al., 1994a,b; Graham et al., 2004; Reichard et al., 2005; Hortobágyi et al., 2007; Johnson et al., 2013, 2016; Koch et al., 2020). Unlike avidin that is not present in mammals, biotin can be up taken from foods and is widely distributed in the brain (Wood and Warnke, 1981; Wang and Pevsner, 1999; McKay et al., 2008). Biotin is enriched in oligodendrocytes, the predominant cells in white matter tracts (LeVine and Macklin, 1988; McKay et al., 2004) and is also present in astrocytes (Xiong et al., 2023, 2024). To determine whether endogenous biotin in these glial cells is the underlying source of the observed varicosities, we directly stained healthy brains with HRP-conjugated avidin (HRP-Avidin) and demonstrated fibrous astrocytes and varicosity-like oligodendrocytes in white matter tracts (Xiong et al., 2023, 2024). Significantly, we found dramatically increased HRP-Avidin staining in injured mice, suggesting an upregulation in endogenous biotin after TBI (Xiong et al., 2023). Given that injury results in astrogliosis (Smith et al., 2015; Shahim et al., 2017) and oligodendrogliosis (Flygt et al., 2016), the varicosities observed with immunoperoxidase staining are therefore most likely due to avidin binding to endogenous biotin in activated glial cells.

Additional support for avidin binding to endogenous biotin as the source of the varicosities comes from the extraordinarily high dilution of the primary antibody 22C11 in immuonperoxidase staining in tissue from TBI patients. While some researchers diluted 22C11 at 1:100–200 (Ryu et al., 2014; Xiong et al., 2023, 2024) as recommended by the manufacturer (Millipore-Sigma), others used this primary antibody at a concentration as high as 1:80,000 or even 1:130,000 (Johnson et al., 2016; Koch et al., 2020). Highly diluted primary antibodies produce very weak signals that are easily masked and overshadowed by spurious staining. Therefore, it is highly likely that the varicosities in white matter tracts observed after TBI and widely considered to be a symbol of DAI, actually originated from reactive astrocytes and oligodendrocytes.

Then, what is the most accurate method to determine axonal damage after TBI? Using C-Terminal antibodies specific for APP (including Y188), it has been demonstrated that axonal damage does indeed result in the accumulation of APP. However, the axonal staining is not in the form of varicosities but in blebs i.e., the proximal ends of the truncated axons adjacent to the parent neuronal cell bodies within or near gray matter (Stone et al., 2000; Singleton et al., 2002; Wang et al., 2011; Xiong et al., 2023, 2024). Using transgenic mice, we have verified co-localization of these Y188-positive blebs with fluorescent tags (Xiong et al., 2023). We also observed varicosity-like punctate staining in white matter tracts with Y188 (Xiong et al., 2023). However, these Y188-stained puncta do not co-localize with damaged axons in transgenic mice after TBI, suggesting that they are not derived from axons, but likely to be originated from oligodendrocytes that express APP (Palacios et al., 1992; Skaper et al., 2009; Xiong et al., 2023). Therefore, C-Terminal antibodies are still useful biomarkers for axonal blebs (or truncation) after TBI, with the caveat that white matter oligodendrocytes are stained in a varicosity-like pattern. It needs to be stated that these C-Terminal antibodies have been tested only in rodents for detecting axonal blebs in gray matter and puncta in white matter after TBI. They should also be applicable for TBI patient samples, as the amino acid sequence of APP is identical between rodents and humans.

While DAI in white matter tracts is prominent after TBI, TBI-induced pathology should not be confined to DAI. Neuronal cell body damage, dendritic deformation and Wallerian degeneration (of axons) have all been demonstrated by staining with Fluoro-Jade dyes (Yang et al., 2015, 2020; Xiong et al., 2023, 2024) and/or the NeuroSilver kit (Koliatsos et al., 2011; Xiong et al., 2023, 2024). These two major makers, together with C-Terminal antibodies for APP (such as Y188) can detect different pathological structures that emerge at different time windows after TBI. We therefore recommend that a combination of different biomarkers should be adopted and different time points need to be checked when assessing neuropathology after TBI (Xiong et al., 2024).

In conclusion, 22C11 is not specific for APP and the varicosities in white matter tracts observed after immunoperoxidase staining may not represent axonal damages, but reactive glial cells. A combination of biomarkers revealing different stages of the injury will provide the most accurate and comprehensive pathology after TBI.

## References

[B1] Adams J. H. Doyle D. Ford I. Gennarelli T. A. Graham D. I. McLellan D. R.. (1989). Diffuse axonal injury in head injury: definition, diagnosis and grading. Histopathology 15, 49–59. 10.1111/j.1365-2559.1989.tb03040.x 2767623

[B2] Adams J. H. Graham D. I. Murray L. S. Scott G. (1982). Diffuse axonal injury due to nonmissile head injury in humans: an analysis of 45 cases. Ann. Neurol. 12, 557–563. 10.1002/ana.410120610 7159059

[B3] Bhattacharjee J. Cardozo B. N. Kamphuis W. Kamermans M. Vrensen G. F. (1997). Pseudo-immunolabelling with the avidin-biotin-peroxidase complex (ABC) due to the presence of endogenous biotin in retinal Müller cells of goldfish and salamander. J. Neurosci. Methods 77, 75–82. 10.1016/S0165-0270(97)00114-3 9402560

[B4] Chauvet N. Apert C. Dumoulin A. Epelbaum J. Alonso G. (1997). Mab22C11 antibody to amyloid precursor protein recognizes a protein associated with specific astroglial cells of the rat central nervous system characterized by their capacity to support axonal outgrowth. J. Comp. Neurol. 377, 550–564. 9007192

[B5] Chen X.-. H Johnson V. E. Uryu H. Trojanowski J. Q. Smith D. H. (2009). A lack of amyloid beta plaques despite persistent accumulation of amyloid beta in axons of long-term survivors of traumatic brain injury. Brain Pathol. 19, 214–223. 10.1111/j.1750-3639.2008.00176.x 18492093 PMC3014260

[B6] Coronado V. G. Xu L. Basavaraju S. V. McGuire L. C. Wald M. M. Faul M. D.. CDC (2011). Surveillance for traumatic brain injury-related deaths—United States, 1997–2007. MMWR. Surveill. Summ. 60, 1–32. 21544045

[B7] Del Turco D. Paul M. H. Schlaudraff J. Hick M. Endres K. Müller U. C.. (2016). Region-specific differences in amyloid precursor protein expression in the mouse hippocampus. Front. Mol. Neurosci. 9:134. 10.3389/fnmol.2016.00134 27965537 PMC5126089

[B8] Flygt J. Clausen F. Marklund N. (2016). Diffuse traumatic brain injury in the mouse induces a transient proliferation of oligodendrocyte progenitor cells in injured white matter tracts. Rest Neurol. Neurosci. 35, 251–263. 10.3233/RNN-160675 27768001

[B9] Gentleman S. M. Nash M. J. Sweeting C. J. Graham D. I. Roberts G. W. (1993). Beta-amyloid precursor protein (beta APP) as a marker for axonal injury after head injury. Neurosci. Lett. 160, 139–144. 10.1016/0304-3940(93)90398-5 8247344

[B10] Gentleman S. M. Roberts G. W. Gennarelli T. A. Maxwell W. L. Adams J. H. Kerr S.. (1995). Axonal injury: a universal consequence of fatal closed head injury? Acta Neuropathol. 89, 537–543. 10.1007/BF00571509 7676809

[B11] Graham D. I. Smith C. Reichard R. Leclercq P. D. Gentleman S. M. (2004). Trials and tribulations of using beta-amyloid precursor protein immunohistochemistry to evaluate traumatic brain injury in adults. Forensic Sci. Int. 146, 89–96. 10.1016/S0379-0738(03)00274-3 15542268

[B12] Guo Q. Li H. Gaddam S. S. Justice N. J. Robertson C. S. Zheng H.. (2012). Amyloid precursor protein revisited: neuron-specific expression and highly stable nature of soluble derivatives. J. Biol. Chem. 287, 2437–2445. 10.1074/jbc.M111.315051 22144675 PMC3268404

[B13] Hånell A. Greer J. E. McGinn M. J. Povlishock J. T. (2015). Traumatic brain injury-induced axonal phenotypes react differently to treatment. Acta Neuropathol. 129, 317–332. 10.1007/s00401-014-1376-x 25528329

[B14] Hayes J. P. Bigler E. D. Verfaellie M. (2016). Traumatic Brain Injury as a Disorder of Brain Connectivity. J. Int. Neuropsychol. Soc. 22, 120–113. 10.1017/S1355617715000740 26888612 PMC5734864

[B15] Hick M. Herrmann U. Weyer S. W. Mallm J. P. Tschäpe J. A. Borgers M.. (2015). Acute function of secreted amyloid precursor protein fragment APPsα in synaptic plasticity. Acta Neuropathol. 129, 21–37. 10.1007/s00401-014-1368-x 25432317

[B16] Hortobágyi T. Wise S. Hunt N. Cary N. Djurovic V. Fegan-Earl A.. (2007). Traumatic axonal damage in the brain can be detected using beta-APP immunohistochemistry within 35 min after head injury to human adults. Neuropathol. Appl. Neurobiol. 33, 226–237. 10.1111/j.1365-2990.2006.00794.x 17359363

[B17] Johnson V. E. Stewart W. Smith D. H. (2013). Axonal pathology in traumatic brain injury. Exp. Neurol. 246, 35–43. 10.1016/j.expneurol.2012.01.013 22285252 PMC3979341

[B18] Johnson V. E. Stewart W. Weber M. T. Cullen D. K. Siman R. Smith D. H.. (2016). SNTF immunostaining reveals previously undetected axonal pathology in traumatic brain injury. Acta Neuropathol. 131, 115–135. 10.1007/s00401-015-1506-0 26589592 PMC4780426

[B19] Koch P. F. Cottone C. Adam C. D. Ulyanova A. V. Russo R. J. Weber M. T.. (2020). Traumatic brain injury preserves firing rates but disrupts laminar oscillatory coupling and neuronal entrainment in hippocampal CA1. eNeuro 7:eNeuro.0495-19.2020. 10.1523/ENEURO.0495-19.2020 32737188 PMC7477953

[B20] Koliatsos V. E. Cernak I. Xu L. Song Y. Savonenko A. Crain B. J.. (2011). A mouse model of blast injury to brain: initial pathological, neuropathological, and behavioral characterization. J. Neuropathol. Exp. Neurol. 70, 399–416. 10.1097/NEN.0b013e3182189f06 21487304

[B21] LeVine S. M. Macklin M. B. (1988). Biotin enrichment in oligodendrocytes in the rat brain. Brain Res. 444, 199–203. 10.1016/0006-8993(88)90930-4 3359289

[B22] McKay B. E. Molineux M. L. Turner R. W. (2004). Biotin is endogenously expressed in select regions of the rat central nervous system. J. Comp. Neurol. 473, 86–96. 10.1002/cne.20109 15067720

[B23] McKay B. E. Molineux M. L. Turner R. W. (2008). Endogenous biotin in rat brain: implications for false-positive results with avidin-biotin and streptavidin-biotin techniques. Methods Mol. Biol. 418, 111–128. 10.1007/978-1-59745-579-4_10 18287654

[B24] Mierzwa A. J. Marion C. M. Sullivan G. M. McDaniel D. P. Armstrong R. C. (2015). Components of myelin damage and repair in the progression of white matter pathology after mild traumatic brain injury. J. Neuropathol. Exp. Neurol. 74, 218–232. 10.1097/NEN.0000000000000165 25668562 PMC4327393

[B25] Müller U. C. Deller T. Korte M. (2017). Not just amyloid: physiological functions of the amyloid precursor protein family. Nat. Rev. Neurosci. 18, 281–298. 10.1038/nrn.2017.29 28360418

[B26] Nolan A. L. Petersen C. Iacono D. Mac Donald C. L. Mukherjee P. van der Kouwe A.. (2021). Tractography-pathology correlations in traumatic brain injury: a TRACK-TBI study. J. Neurotrauma 38, 1620–1631. 10.1089/neu.2020.7373 33412995 PMC8165468

[B27] Palacios G. Palacios J. M. Mengod G. Frey P. (1992). Beta-amyloid precursor protein localization in the Golgi apparatus in neurons and oligodendrocytes. An immunocytochemical structural and ultrastructural study in normal and axotomized neurons. Brain Res. Mol. Brain Res 15, 195–206. 10.1016/0169-328X(92)90109-O 1331676

[B28] Reichard R. R. Smith C. Graham D. I. (2005). The significance of beta-APP immunoreactivity in forensic practice. Neuropathol. Appl. Neurobiol. 31, 304–313. 10.1111/j.1365-2990.2005.00645.x 15885067

[B29] Ryu J. Horkayne-Szakaly I. Xu L. Pletnikova O. Leri F. Eberhart C.. (2014). The problem of axonal injury in the brains of veterans with histories of blast exposure. Acta Neuropathol. Commun. 2:153. 10.1186/s40478-014-0153-3 25422066 PMC4260204

[B30] Shahim P. Tegner Y. Marklund N. Höglund K. Portelius E. Brod D. L.. (2017). Astroglial activation and altered amyloid metabolism in human repetitive concussion. Neurology 88, 1400–1407. 10.1212/WNL.0000000000003816 28283595 PMC5386435

[B31] Sherriff F. E. Bridges L. R. Gentleman S. M. Sivaloganathan S. Wilson S. (1994a). Markers of axonal injury in post mortem human brain. Acta Neuropathol. 88, 433–439. 10.1007/BF00389495 7847072

[B32] Sherriff F. E. Bridges L. R. Sivaloganathan S. (1994b). Early detection of axonal injury after human head trauma using immunocytochemistry for beta-amyloid precursor protein. Acta Neuropathol. 87, 55–62. 10.1007/BF00386254 8140894

[B33] Singleton R. H. Zhu J. Stone J. R. Povlishock J. T. (2002). Traumatically induced axotomy adjacent to the soma does not result in acute neuronal death. JNS 22, 791–802. 10.1523/JNEUROSCI.22-03-00791.2002 11826109 PMC6758486

[B34] Skaper S. D. Evans N. A. Soden P. E. Rosin C. Facci L. Richardson J. C.. (2009). Oligodendrocytes are a novel source of amyloid peptide generation. Neurochem Res. 34, 2243–2250. 10.1007/s11064-009-0022-9 19557514

[B35] Smith C. J. Xiong G. Elkind J. A. Putnam B. Cohen A. S. (2015). Brain injury impairs working memory and prefrontal circuit function. Front. Neurol. 6:240. 10.3389/fneur.2015.00240 26617569 PMC4643141

[B36] Stone J. R. Singleton R. H. Povlishock J. T. (2000). Antibodies to the C-terminus of the b-amyloid precursor protein (APP): a site specific marker for the detection of traumatic axonal injury. Brain Res. 871, 288–302. 10.1016/S0006-8993(00)02485-9 10899295

[B37] Wang H. Pevsner J. (1999). Detection of endogenous biotin in various tissues: novel functions in the hippocampus and implications for its use in avidin-biotin technology. Cell Tissue Res. 296, 511–516. 10.1007/s004410051311 10370137

[B38] Wang J. Hamm R. J. Povlishock J. T. (2011). Traumatic axonal injury in the optic nerve: evidence for axonal swelling, disconnection, dieback, and reorganization. J. Neurotrauma 28, 1185–1198. 10.1089/neu.2011.1756 21506725 PMC3136743

[B39] Wood G. S. Warnke R. (1981). Suppression of endogenous avidin-binding activity in tissues and its relevance to biotin-avidin detection systems. J. Histochem. Cytochem. 29, 1196–1204. 10.1177/29.10.7028859 7028859

[B40] Xiong G. Jean I. Farrugia A. M. Metheny H. Johnson B. N. Cohen N. A.. (2024). Temporal and structural sensitivities of major biomarkers for detecting neuropathology after traumatic brain injury in the mouse. Front. Neursci. 18:1339262. 10.3389/fnins.2024.1339262 38356651 PMC10865493

[B41] Xiong G. Metheny H. Hood K. Jean I. Farrugia A. M. Johnson B. N.. (2023). Detection and verification of neurodegeneration after traumatic brain injury in the mouse: Immunohistochemical staining for amyloid precursor protein. Brain Pathol. 33:e13163. 10.1111/bpa.13163 37156643 PMC10580020

[B42] Yang L.-. Y Chu Y-, H. Tweedie D. Yu Q.-. S.. (2015). Post-trauma administration of the pifithrin-α oxygen analog improves histological and functional outcomes after experimental traumatic brain injury. Exp. Neurol. 269, 56–66. 10.1016/j.expneurol.2015.03.015 25819102 PMC5193498

[B43] Yang L. Y. Greig N. H. Tweedie D. Jung Y. J. Chiang Y. H. Hoffer B. J.. (2020). (2020). The p53 inactivators pifithrin-μ and pifithrin-α mitigate TBI-induced neuronal damage through regulation of oxidative stress, neuroinflammation, autophagy and mitophagy. Exp. Neurol. 324:113135. 10.1016/j.expneurol.2019.113135 31778663 PMC7792017

[B44] Yasuoka K. Hirata K. Kuraoka A. He J. Kawabuchi M. (2004). Expression of amyloid precursor protein-like molecule in astroglial cells of the subventricular zone and rostral migratory stream of the adult rat forebrain. J. Anatomy 205, 135–146. 10.1111/j.0021-8782.2004.00320.x 15291796 PMC1571331

[B45] Young M. J. Lee R. K. Jhaveri S. Wurtman R. J. (1999). Intracellular and cell surface distribution of amyloid precursor protein in cortical astrocytes. Brain Res. Bull. 50, 27–32. 10.1016/S0361-9230(99)00084-2 10507468

[B46] Zempleni J. Wijeratne S. S. K. Hassan Y. I. (2009). Biotin. Biofactors 35, 36–46. 10.1002/biof.8 19319844 PMC4757853

[B47] Ziogas N. K. Koliatsos V. E. (2018). Primary Traumatic Axonopathy in Mice Subjected to Impact Acceleration: A Reappraisal of Pathology and Mechanisms with High-Resolution Anatomical Methods. JNS 38:4031–4047. 10.1523/JNEUROSCI.2343-17.2018 29567804 PMC6705930

